# Complete Genome Sequences of *Massilia* sp. Strains B-10 and H-1, Isolated from the Water in the Shulgan-Tash Cave

**DOI:** 10.1128/mra.01160-22

**Published:** 2023-01-12

**Authors:** Anna Sazanova, Andrey Belimov, Yuri Gogolev, Elizaveta Chirak, Alexey Afonin, Denis Karlov, Irina Kuznetsova, Polina Guro, Lyudmila Kuzmina, Vera Safronova

**Affiliations:** a All-Russia Research Institute for Agricultural Microbiology, St. Petersburg, Russian Federation; b Kazan Institute of Biochemistry and Biophysics, Federal Research Center Kazan Scientific Center of the Russian Academy of Sciences, Kazan, Russian Federation; c Ufa Institute of Biology, Ufa Federal Research Center, Russian Academy of Sciences, Ufa, Russian Federation; Wellesley College

## Abstract

In this article, we report the complete genome sequences of *Massilia* sp. strains B-10 (RCAM05335) and H-1 (RCAM05339), which were isolated from the water of the Dal’nee Verkhnee Lake in the Shulgan-Tash cave in Russia (53°2′0″N, 57°3′0″E). The sequences were obtained using an Oxford Nanopore Technologies MinION system.

## ANNOUNCEMENT

The Dal’nee Verkhnee Lake is located in an extremely inaccessible part of the Shulgan-Tash cave and has calcium bicarbonate-type water, with total mineralization of 390 to 510 mg/L (1). The study of the microbiome of this ecological niche is important for understanding the metabolic relationships among chemoautotrophic microorganisms. Forty-milliliter water samples from the surface layer of the lake were centrifuged for 15 min at 15,000 rpm. The pellet was resuspended in 0.5 mL of sterile tap water and distributed on Reasoner's 2A (R2A) agar medium diluted 10 times. The isolate formed visible colonies after 7 days of incubation at 6°C; cultures were purified by cloning and stored at −80°C.

Genomic DNA was extracted and purified from individual colonies using the DNeasy blood and tissue kit (Qiagen, Germany) according to the recommendations of the manufacturer. Long-read whole-genome sequencing was performed using a MinION sequencer with an R9.4.1 rev D flow cell (Oxford Nanopore Technologies, England) in the Core Centrum Genomic Technologies, Proteomics, and Cell Biology at the All-Russia Research Institute for Agricultural Microbiology (ARRIAM). Isolated DNA without size selection was used with the SQK-LSK109 ligation sequencing kit, and the EXP-NBD104 and EXP-NBD114 native barcoding expansion kits were used to prepare the library according to the manufacturer’s instructions. Barcode 7 was used for these genomes; 429.4 Mb and 57,676 reads were generated for strain RCAM05335, and 1.2 Gb and 325,182 reads were generated for strain RCAM05339. The reads were base called and demultiplexed and the barcodes were trimmed using the Guppy base caller v5.0.11. The Flye assembler v2.8 was used to assemble the Nanopore reads (2). The resulting assembly was corrected four times using Racon v1.3.2 (3) (parameters: -m 8 -x -6 -g -8 -w 500), followed by a single polish using the Medaka v1.4.3 program (https://github.com/nanoporetech/medaka) with default parameters. The quality of the assemblies was evaluated with QUAST v5.0.2 (4). General features of genome sequencing and assembly of *Massilia* sp. strains RCAM05335 and RCAM05335 are presented in Table 1. The search for genes in the assembled contigs was performed using the NCBI Prokaryotic Genome Annotation Pipeline (PGAP) v5.3 (5). The circularity of all of the assembled fragments was reported by the Flye assembler and verified by mapping the long reads to the assembled fragments using Minimap2 (6) with the map-ont mapping mode and inspecting the coverage uniformity; no specific starting point was chosen for the assembled contigs.

**TABLE 1:** General features of genome sequencing and assembly of *Massilia* sp. strains RCAM05335 and RCAM05335

Genome	GenBank accession no.	BioSample accession no.	SRA accession no.	Genome size (bp)	GC content (%)	Genome coverage (×)	No. of coding sequences	Most closely related to type strain	Similarity of *rrs* gene to type strain sequence (%)
RCAM05335	CP087970	SAMN23078439	SRR21050825	5,833,599	63.35	68	8,249	Massilia violaceinigra B2	99.28
RCAM05339	CP087969	SAMN23078438	SRR21050826	5,836,689	63.41	183	8,116	Massilia violaceinigra B2	99.41

The 16S rRNA (*rrs*) gene sequences of strains RCAM05335 and RCAM05339 were compared with those of type strains available in the GenBank database using BLAST analysis. These strains are most closely related to the type strain Massilia violaceinigra B2 (GenBank accession number NR_178603.1) and belong to a new species of the genus *Massilia* (Table 1).

### Data availability.

The complete genome sequences were deposited in GenBank under accession numbers CP087970 and CP087969, corresponding to the BioSample accession numbers SAMN23078439 and SAMN23078438, and the raw reads were deposited under SRA accession numbers SRR21050825 and SRR21050826.

## References

[1] Chervyatsova OY, Potapov SS, Kuz’mina LY, Leonova LV. 2018. Subaqueous stalactoids in the Dal’nee Verkhnee lake of the Shulgan-Tash cave (southern Urals). News Ural State Mining Univ 2:20–25. doi:10.21440/2307-2091-2018-2-20-25.

[2] Kolmogorov M, Yuan J, Lin Y, Pevzner P. 2019. Assembly of long error-prone reads using repeat graphs. Nat Biotechnol 37:540–546. doi:10.1038/s41587-019-0072-8. 30936562

[3] Vaser R, Sović I, Nagarajan N, Šikić M. 2017. Fast and accurate de novo genome assembly from long uncorrected reads. Genome Res 27:737–746. doi:10.1101/gr.214270.116. 28100585 PMC5411768

[4] Mikheenko A, Prjibelski A, Saveliev V, Antipov D, Gurevich A. 2018. Versatile genome assembly evaluation with QUAST-LG. Bioinformatics 34:i142–i150. doi:10.1093/bioinformatics/bty266. 29949969 PMC6022658

[5] Li W, O'Neill KR, Haft DH, DiCuccio M, Chetvernin V, Badretdin A, Coulouris G, Chitsaz F, Derbyshire MK, Durkin AS, Gonzales NR, Gwadz M, Lanczycki CJ, Song JS, Thanki N, Wang J, Yamashita RA, Yang M, Zheng C, Marchler-Bauer A, Thibaud-Nissen F. 2021. RefSeq: expanding the Prokaryotic Genome Annotation Pipeline reach with protein family model curation. Nucleic Acids Res 49:D1020–D1028. doi:10.1093/nar/gkaa1105. 33270901 PMC7779008

[6] Li H. 2018. Minimap2: pairwise alignment for nucleotide sequences. Bioinformatics 34:3094–3100. doi:10.1093/bioinformatics/bty191. 29750242 PMC6137996

